# Oral Administration of N-Acetyl-seryl-aspartyl-lysyl-proline Ameliorates Kidney Disease in Both Type 1 and Type 2 Diabetic Mice via a Therapeutic Regimen

**DOI:** 10.1155/2016/9172157

**Published:** 2016-03-20

**Authors:** Kyoko Nitta, Sen Shi, Takako Nagai, Megumi Kanasaki, Munehiro Kitada, Swayam Prakash Srivastava, Masakazu Haneda, Keizo Kanasaki, Daisuke Koya

**Affiliations:** ^1^Department of Diabetology & Endocrinology, Kanazawa Medical University, Uchinada, Ishikawa 920-0293, Japan; ^2^Medical Research Institute, Kanazawa Medical University, Uchinada, Ishikawa 920-0293, Japan; ^3^Division of Metabolism and Biosystemic Science, Department of Medicine, Asahikawa Medical University, Midorigaoka Higashi 2-1-1-1, Asahikawa 078-8510, Japan

## Abstract

Kidney fibrosis is the final common pathway of progressive kidney diseases including diabetic nephropathy. Here, we report that the endogenous antifibrotic peptide N-acetyl-seryl-aspartyl-lysyl-proline (AcSDKP), the substrate of angiotensin-converting enzyme (ACE), is an orally available peptide drug used to cure kidney fibrosis in diabetic mice. We utilized two mouse models of diabetic nephropathy, streptozotocin- (STZ-) induced type 1 diabetic CD-1 mice and type 2 diabetic nephropathy model* db/db* mice. Intervention with the ACE inhibitor imidapril, oral AcSDKP, or imidapril + oral AcSDKP combination therapy increased urine AcSDKP levels. AcSDKP levels were significantly higher in the combination group compared to those of the other groups. AcSDKP oral administration, either AcSDKP alone or in addition to imidapril, ameliorated glomerulosclerosis and tubulointerstitial fibrosis. Plasma cystatin C levels were higher in both models, at euthanasia, and were restored by all the treatment groups. The levels of antifibrotic miRs, such as miR-29 or let-7, were suppressed in the kidneys of both models; all treatments, especially the combination of imidapril + oral AcSDKP, restored the antifibrotic miR levels to a normal value or even higher. AcSDKP may be an oral antifibrotic peptide drug that would be relevant to combating fibroproliferative kidney diseases such as diabetic nephropathy.

## 1. Introduction

Diabetic nephropathy is the most common cause of kidney disease and causes significant health problems [1, 2]. The most common therapy for diabetic nephropathy is based on renin-angiotensin system (RAS) inhibitors [3–5]. Indeed, RAS inhibitors have been shown to exhibit renal protective effects; the residual risks in the progression of diabetic nephropathy represented major burdens [1, 2]. Therefore, in addition to established therapies for diabetic nephropathy, such as appropriate blood glucose control and RAS inhibitor use, an alternative novel therapy to combat diabetic nephropathy is urgently needed [1, 2].

Among RAS inhibitors, angiotensin-converting enzyme inhibitors (ACE-I) and angiotensin II type 1 receptor blockers (ARB) are two major drug classes prescribed to delay diabetic nephropathy progression [6, 7]. These two drug classes may exhibit similar renoprotective effects but also may display significant differences in organ protection from diabetes-associated damage [7–11]. It is well known that the maximum dose of ACE-I, which abolishes circulatory angiotensin II, cannot inhibit local angiotensin II production in renal tubules [12]. The ACE-I captopril provides therapeutic effects to adriamycin-induced nephropathy and kidney fibrosis in mice; however, ARB losartan and ARB plus statin therapy did not ameliorate the kidney pathology [13]. Recently, we reported that the ACE-I imidapril did but ARB TA-606 did not suppress kidney fibrosis in streptozotocin-induced diabetic CD-1 mice [14]. A recent meta-analysis revealed that ACE-I exhibited stronger organ protection compared to ARB in patients with type 2 diabetes with nephropathy [9, 11]. These reports suggested that the biology of ACE in kidney fibrosis is not limited to “angiotensin-conversion” but may involve some angiotensin-independent effects.

There are two catalytic domains, N-terminal and C-terminal, in the ACE [7]. Angiotensin I is mainly metabolized into angiotensin II at the C-terminal catalytic site of ACE; the N-terminal specific substrate N-acetyl-seryl-aspartyl-lysyl-proline (AcSDKP) is the antifibrotic peptide [15] synthesized through protein processing, which is generated by prolyl oligopeptidase from the precursor peptide thymosin *β*4 [15]. Plasma AcSDKP is at its minimal level in the basal condition; the administration of ACE-I (captopril) has been shown to increases the concentration of plasma AcSDKP fivefold [15, 16].

We and others have shown that AcSDKP inhibits transforming growth factor- (TGF-) *β*-induced fibrogenic gene expression in human mesangial cells [17] or fibroblasts [18] by inhibiting Smad2/3 signaling. AcSDKP ameliorated glomerular damage in* db/db* mice [19] and in STZ-induced diabetic CD-1 mice [14]. AcSDKP displayed antifibrotic and organ protective effects in various experimental models [20–28]. Recently, we showed that the combination therapy of ACE-I with AcSDKP displayed strong antifibrotic effects via the induction of microRNA (miR) let-7 and inhibition of the endothelial-mesenchymal transition [14]. Additionally, AcSDKP suppressed platelet-derived growth factor- (PDGF-) B-induced glomerular mesangial proliferation [29]. These reports clearly demonstrate that AcSDKP could be a potential drug to combat kidney fibrosis in diabetes. In this study, we aim to investigate whether the oral administration of AcSDKP can increase its plasma or urine concentration to a therapeutic range and exhibit protective effects in diabetic mice with kidney fibrosis.

## 2. Materials and Methods

### 2.1. Materials

The AcSDKP was a kind gift from Dr. Omata from Asabio Bio Technology (Osaka, Japan). Imidapril (ACE-I) was provided by Mitsubishi Tanabe Pharma (Osaka, Japan) via an MTA agreement.

### 2.2. Animal Experiments

All in vivo experiments were performed according to the institutional ethical committee guidelines (protocol number: 2014-101). For the streptozotocin- (STZ-) induced type 1 diabetic animal model, we utilized CD-1, a mouse strain that displays severe diabetic-associated fibrosis [14, 30–32]. Eight-week-old male CD-1 mice (Sankyo lab service, Tokyo, Japan) were administered a single intraperitoneal injection of STZ (200 mg/kg); control mice were injected with citrate buffer. Two weeks after the STZ injection, the mice with blood glucose levels > 16 mM were confirmed as valid diabetes models. We initiated intervention with ACE-I imidapril from 16 weeks after induction of diabetes in CD-1 mice. Four weeks after imidapril intervention, some diabetic CD-1 mice were initiated with AcSDKP (1 mg/day) oral administration 3 times per week for 1 month.* Db/db* (*BKS. Cg-Dock7*〈*m*〉*+/+Lepr*〈*db*〉*/J*) or control* db/m* mice were obtained from the Jackson Laboratory (Bar Harbor, Maine). At 20 weeks of age, the* db/db* mice were divided into the following four groups: imidapril [2.5 mg/kg BW/day], oral AcSDKP [1 mg/day 3 times/week], oral AcSDKP + imidapril, and the control group. Imidapril was provided in the drinking water. All the* db/db* or* db/m* mice were euthanized at 24 weeks of age. Plasma cystatin C measurements were performed using an ELISA kit (R&D Systems, Minneapolis, MN). Urine albumin levels were estimated using a Mouse Albumin ELISA Kit (Exocell, Philadelphia, PA**)**.

### 2.3. AcSDKP Measurements

Blood was harvested into a heparinized tube containing captopril (final concentration 10 *μ*mol/L) and centrifuged at 3,000 ×g for 15 min at 4°C. The plasma was kept in a −80°C freezer until the following assays were performed. We estimated plasma and urine AcSDKP concentrations using a competitive enzyme immunoassay kit (SPI-BIO, Massy, France) according to the manufacturer's instructions.

For the collection of urine to estimate AcSDKP levels, we harvested urine before, and 2, 24, 48 hours after the final oral administration of AcSDKP at 24 weeks of age (*db/db*) or at 24 weeks after the initiation of diabetes (CD-1 mice).

### 2.4. Morphological Evaluation

We utilized a point-counting method to evaluate the relative area of the mesangial matrix. We analyzed 10 PAS-stained glomeruli from each mouse using a digital microscope screen grid containing 540 (27 × 20) points and employing Adobe Photoshop Elements 6.0. The number of grid points on the mesangial matrix (eosin positive area) was divided by the total number of points in each glomerulus to calculate the mesangial matrix area as the percentage of the total grid of the glomerulus. Masson's trichrome-stained tissue images were evaluated by ImageJ software, and the fibrotic areas were estimated. For each mouse, images of six different fields of view were evaluated at 100x magnification.

### 2.5. Western Blot Analysis

The protein lysates were denatured by boiling in sodium dodecyl sulfate (SDS) sample buffer at 94°C for 5 min. After centrifugation (15,000 ×g for 10 min at 4°C), the proteins in the supernatants were separated on SDS-polyacrylamide gels and transferred onto PVDF membranes (Pall Corporation, Pensacola, FL, USA) via the semidry method. After blocking with TBS-T (Tris-buffered saline containing 0.05% Tween 20) containing 5% bovine serum albumin (BSA), we incubated the membranes with the each primary antibody of target molecules [1 : 1,000 for fibronectin (Novus Biologicals, Littleton, CO, USA), 1 : 100 for FSP-1 (BioLeged, San Diego, CA, USA), and 1 : 500 for *α*SMA (Abcam, Cambridge, UK)] at 4°C overnight. The membranes were washed three times and incubated with each diluted (1 : 2,500) horseradish peroxide (HRP) conjugated-secondary antibody (Cell Signaling Technology, Danvers, MA, USA) for 1 h at room temperature. The immunoreactive bands were developed using an enhanced chemiluminescence (ECL) detection system (Pierce Biotechnology, Rockford, IL, USA) and detected using an ImageQuant LAS 400 digital biomolecular imaging system (GE Healthcare Life Sciences, Uppsala, Sweden).

### 2.6. miR Isolation and qPCR

The kidney samples from each group (control, diabetes, diabetes on imidapril, and diabetes on imidapril with AcSDKP) that had been maintained at −70°C were first incubated in RNA*later* ^*R*^-ICE (Life Technologies) for 16 h at −20°C before homogenization to allow us to extract high-quality miR. Subsequently, tissues were homogenized on ice using a Power Masher (Nippi, Tokyo, Japan). The miRs were extracted using a miRNeasy Mini kit (Qiagen) according to the manufacturer's instructions. Complementary DNA (cDNA) was generated by a miScript II RT kit (Qiagen) using the hiSpec buffer method. miR expression was quantified using a miScript SYBR Green PCR Kit (Qiagen), with 3 ng of complementary DNA. The primers for quantifying Mm_miR-29a, Mm_miR-29b, and Mm_miR-29c were miScript primer assays predesigned by Qiagen. The mature miR sequences were 5′ UAGCACCAUCUGAAAUCGGUUA for Mm_miR-29a, 5′ UAGCACCAUUUGAAAUCAGUGUU for Mm_miR-29b, and 5′ UAGCACCAUUUGAAAUCGGUUA for Mm_miR-29c. All experiments were performed in triplicate, and Hs_RNU6-2_1 (Qiagen) was utilized as an internal control. The primers to quantify Mm_miR-let7b-1, Mm_miR-let7c, and Mm_miR-let7f were designed by Qiagen. The mature sequences were UGGAAGACUUGUGAUUUUGUUGU for Mm_miR-let7b-1, CUGUACAACCUUCUAGCUUUCC for Mm_miR-let7c, and CUAUACAAUCUAUUGCCUUCCC for Mm_miR-let7f, respectively.

### 2.7. Statistical Analysis

The data are expressed as the mean ± SEM values. A one-way ANOVA followed by Tukey's test was used to determine the significance of the differences. Statistical significance was defined as *P* < 0.05. GraphPad Prism software (ver. 5.0f) was used for the statistical analyses.

## 3. Results

### 3.1. Elevation of Both Plasma and Urine AcSDKP Levels after the Oral Administration of AcSDKP

**Figure 1:**
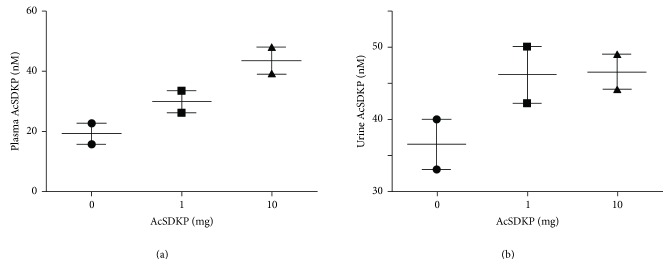
Oral administration of AcSDKP increased the plasma and urine levels of AcSDKP. 8-week-old CD-1 mice were provided single administration of the indicated amount of AcSDKP by oral gavage using a cannula. Two hours after the administration of AcSDKP, the mice were euthanized. (a) Plasma and (b) urine levels of AcSDKP were evaluated by commercially available ELISA kits. *N* = 2 in each group.

Preliminarily, we tried to investigate whether oral administration of antifibrotic peptide AcSDKP can be absorbed from the gastrointestinal tract and increase the plasma levels. We utilized the normal 8-week CD-1 mouse in this experiment and confirmed that, at 2 hours after the oral administration of AcSDKP (1 mg or 10 mg), we found a dose-dependent increase in the levels of AcSDKP in the plasma (Figure 1(a)). We also confirmed that the urinary excretion of AcSDKP was higher in mice administered either 1 mg or 10 mg of AcSDKP compared to PBS-treated mice (Figure 1(b)). These data motivated us to test whether the oral administration of AcSDKP can be used to treat kidney fibrosis in diabetes.

**Figure 2:**
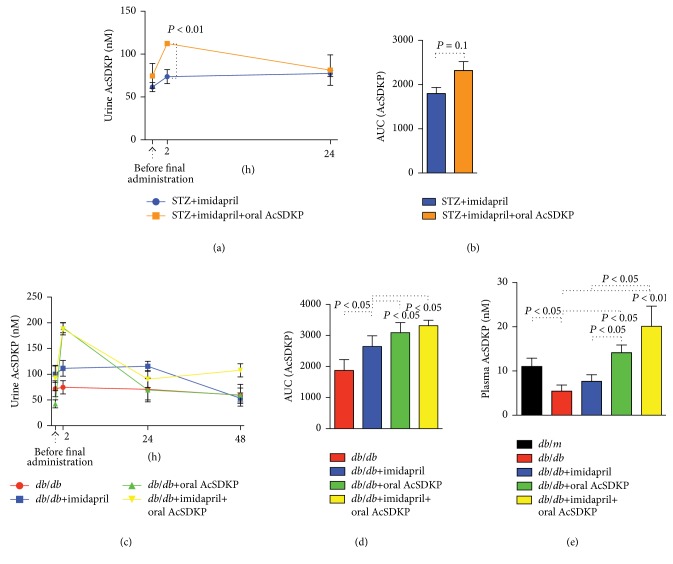
Change in the levels of AcSDKP after oral administration of AcSDKP in diabetic mice. ((a)-(b)) STZ-induced type 1 diabetic mice were treated with imidapril at 16 weeks after the diabetic induction. At 20 weeks after the induction of diabetes, some imidapril-treated mice were given 1 mg of AcSDKP by oral gavage 3 times per week. Urine was harvested just before, after 2 and 24 hours after the final administration of AcSDKP. (a) Time course, (b) area under the curve. *N* = 3 in each group. ((c)-(d)) At 20 weeks of age, diabetic* db/db* mice were divided into 4 groups (no treatment, imidapril, AcSDKP oral, and imidapril + AcSDKP combination). 1 mg of AcSDKP was given by oral gavage 3 times per week. Urine samples were harvested at before, 2 hours, 24 hours, and 48 hours (same as at euthanasia) after the final administration of AcSDKP. (c) Time course, (d) area under the curve. *N* = 5 or 6 in each group were analyzed. (e) Plasma levels of AcSDKP in* db/m* or* db/db* mice at euthanasia. *N* = 5 in each group were analyzed. The data are expressed as the mean ± SEM in the figure.

We utilized two models of diabetic mice to confirm whether the oral administration of AcSDKP inhibits fibrotic changes in the diabetic kidney. First, we used the STZ-induced type 1 model of diabetic CD-1 mice, a well-known fibrotic diabetic kidney disease model [14, 30–32]. We analyzed the urine levels of AcSDKP before the final AcSDKP administration, at 2 hours and at 24 hours after the AcSDKP was given in the imidapril-treated or imidapril + AcSDKP groups. We found that the oral administration of AcSDKP in addition to imidapril further increased AcSDKP urine levels by approximately 1.5-fold at 2 hours after AcSDKP oral administration compared to imidapril alone (Figures 2(a) and 2(b)). The plasma levels of AcSDKP were not different between the imidapril and imidapril + AcSDKP groups at euthanasia in the STZ-induced diabetic CD-1 mice (data not shown: after 48 hours of final administration of AcSDKP).

Next, we analyzed the* db/db* mice, the mouse model of type 2 diabetes.* Db/db* mice at 20 weeks old were given vehicle, imidapril, AcSDKP, or an imidapril + AcSDKP combination therapy. Imidapril treatment significantly increased the urine AcSDKP concentration compared to vehicle alone in the* db/db* mice (Figures 2(c) and 2(d)). AcSDKP oral administration alone transiently increased urine levels of AcSDKP, but they returned to basal levels by 24 hours after administration, and the levels were similar to those of the mice treated with imidapril alone (Figures 2(c) and 2(d)). The imidapril + AcSDKP combination treatment exhibited a similar peak concentration of urine AcSDKP as AcSDKP alone; however, the levels of AcSDKP in the urine at euthanasia were significantly higher in the combination treatment compared to that of the other groups (Figure 2(c)). AcSDKP is the substrate of ACE; therefore, it is reasonable to confirm that the combination therapy exhibited higher levels of AcSDKP in urine. When euthanizing the mice, we harvested the plasma and found that the plasma levels of AcSDKP were significantly lower in the* db/db* mice compared to those of the* db/m* mice (Figure 2(e)). Imidapril alone did not significantly increase the plasma concentration of AcSDKP; treatment with AcSDKP alone increased plasma levels, and the plasma levels of AcSDKP in combination (imidapril and AcSDKP) were much higher (at 48 hours after the final administration of AcSDKP).

### 3.2. A Higher Level of AcSDKP was Associated with Antifibrotic Effects in the Kidney

We have shown that AcSDKP can inhibit kidney fibrosis in STZ-induced diabetic CD-1 mice [14] or mesangial expansion in* db/db* mice [19], independent of blood pressure or blood glucose levels [14, 19]. Similar to a previous study, all STZ-induced diabetic CD-1 mice weighed less than the nondiabetic mice (see Supplemental Figure 1 in Supplementary Material available online at http://dx.doi.org/10.1155/2016/9172157). Additionally, the kidney per body weight was heavier in diabetic mice compared to that of the control mice (Supplemental Figure 1). None of the treatments altered this trend in CD-1 diabetic mice (Supplemental Figure 1). Blood glucose was not altered by the treatment (Supplemental Figure 1). In addition, the* db/db* mice weighed more than the control* db/m* mice, and the kidney per body weight was lighter in the diabetic mice compared to that of the control db/m mice (Supplemental Figure 2). Blood glucose levels were higher in the* db/db* mice compared to those of the* db/m* mice (Supplemental Figure 2). These alterations in the* db/db* mice were not affected by any of the treatments (Supplemental Figure 2).

**Figure 3:**
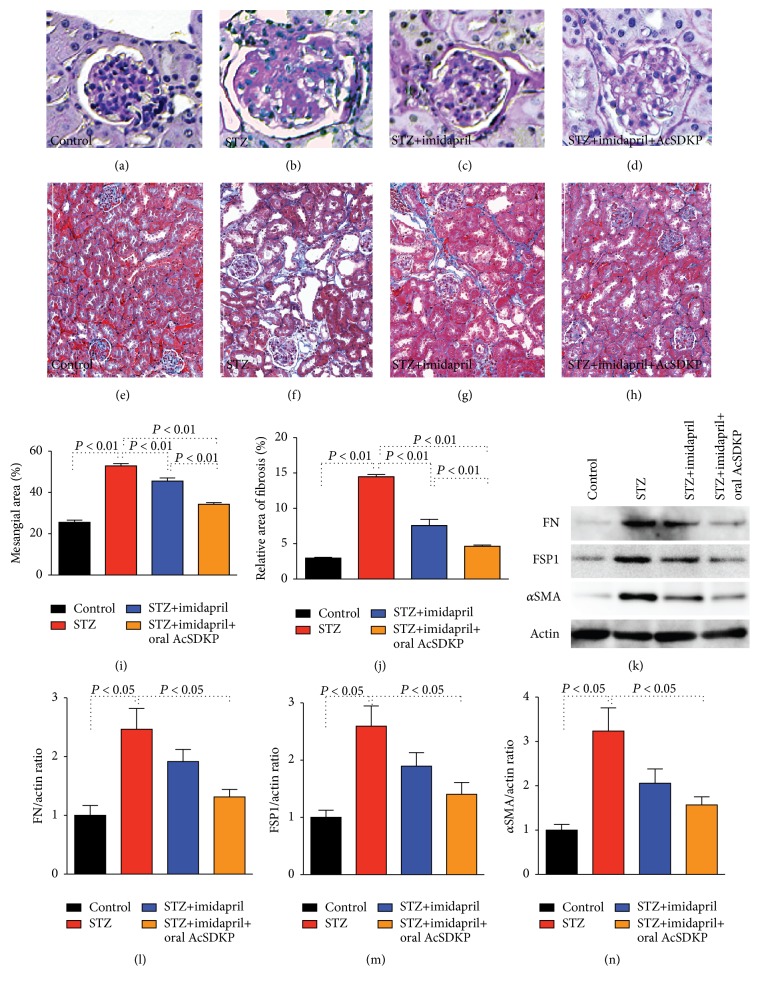
AcSDKP ameliorated glomerulosclerosis and tubulointerstitial fibrosis in STZ-induced diabetes in CD-1 mice. 8-week-old male CD-1 mice were given a single peritoneal injection of STZ. 16 weeks after the induction of diabetes in the CD-1 mice, some diabetic mice were initiated with imidapril in the drinking water. 20 weeks after the induction of diabetes, the imidapril-treated diabetic mice were divided into groups receiving oral gavage of either vehicle or AcSDKP (1 mg) 3 times per week. At 24 weeks after the induction of diabetes, all mice were euthanized. ((a)–(d)) PAS staining for glomeruli and ((e)–(h)) MTS staining for tubulointerstitial fibrosis in the indicated group. Morphometric analyses for mesangial expansion (i) relative area of fibrosis (j) are also shown. (k) Western blot analysis data of indicated proteins. Three mice were analyzed in each group and representative pictures were shown in the figure. Imidapril treatment is labeled as imidapril, and oral AcSDKP treatment is labeled as AcSDKP. ((l)–(n)) Quantitative analysis of indicated proteins to actin expression ratio. Densitometric analysis was performed by ImageJ software. *N* = 3. The data are expressed as the mean ± SEM in the figure. FN: fibronectin.

As shown by Sugimoto et al. [30], at 24 weeks after STZ injection, diabetic CD-1 mice exhibited the accumulation of a PAS-positive matrix in the glomeruli (Figures 3(a), 3(b), and 3(i)). The mesangial area expansion was partially inhibited by imidapril alone, and the diabetic mice treated with the imidapril + AcSDKP combination therapy displayed a remarkable suppression of mesangial expansion (Figures 3(b)–3(d) and 3(i)). Diabetic CD-1 mice exhibited severe tubulointerstitial fibrosis revealed by MTS staining compared to the control mice (Figures 3(e), 3(f), and 3(j)) [30]. Imidapril partially inhibited kidney fibrosis; imidapril + AcSDKP administration provided stronger antifibrotic effects in diabetic CD-1 mice (Figures 3(e)–3(h) and 3(j)). Western blot analysis revealed that diabetic CD-1 mice displayed stronger induction of fibronectin, FSP-1, and *α*SMA protein levels when compared to the control CD-1 mice. Imidapril alone partially and imidapril + AcSDKP combination therapy significantly suppressed such diabetic induced fibronectin, FSP-1, and *α*SMA proteins expression (Figures 3(k)–3(n)).

**Figure 4:**
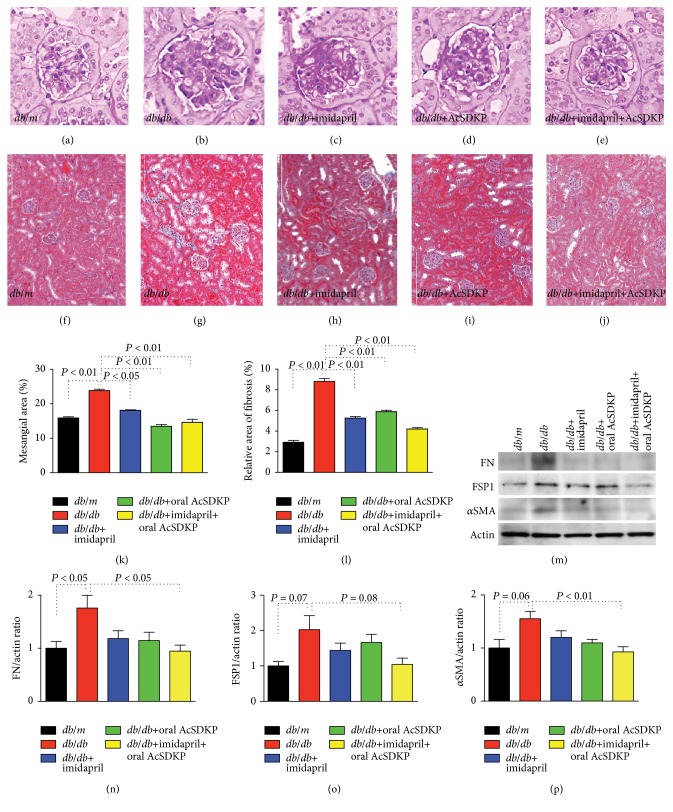
AcSDKP ameliorated kidney damage in* db/db* mice. At 20 weeks of age, the* db/db* mice were divided into the following 4 groups: imidapril, oral AcSDKP, oral AcSDKP + imidapril, and no treatment. AcSDKP (1 mg) was given 3 times per week by oral gavage. At 24 weeks of age, all mice were euthanized. ((a)–(e)) PAS staining for glomeruli and ((f)–(j)) MTS staining for tubulointerstitial fibrosis in the indicated group. Morphometric analyses for mesangial expansion (k) and relative area of fibrosis (l) are also shown. (m) Western blot analysis data of indicated proteins. Three mice were analyzed in each group and representative pictures were shown in the figure. Imidapril treatment is labeled as imidapril, and oral AcSDKP treatment is labeled as AcSDKP. ((n)–(p)) Quantitative analysis of indicated proteins to actin expression ratio. Densitometric analysis was performed by ImageJ software. *N* = 3. The data are expressed as the mean ± SEM in the figure. FN: fibronectin.

*Db/db* mice exhibited expansion of the glomerular mesangial area compared to the control* db/m* mice (Figures 4(a), 4(b), and 4(k)). Imidapril and oral AcSDKP alone displayed significant suppression of glomerular mesangial area expansion; the imidapril + AcSDKP combination therapy exhibited significant suppression of glomerular mesangial expansion in the* db/db* mice (Figures 4(b)–4(e) and 4(k)). Some tubulointerstitial fibrosis was also detected in 24-week-old* db/db* mice compared to the* db/m* mice. The fibrotic area in the* db/db* mice was not found in all intervention groups of* db/db* mice (Figures 4(f)–4(j) and 4(l)). When compared to db/m mice, untreated* db/db* mice exhibited induction of fibronectin protein levels; combination of imidapril + AcSDKP significantly inhibited such induction of fibronectin in* db/db* mice (Figures 4(m) and 4(n)). FSP-1 and *α*SMA protein levels displayed similar trend as fibronectin (Figures 4(m), 4(o), and 4(p)).

### 3.3. Oral AcSDKP Treatment Increased Antifibrotic miR Expression in Diabetic Mice Kidney

**Figure 5:**
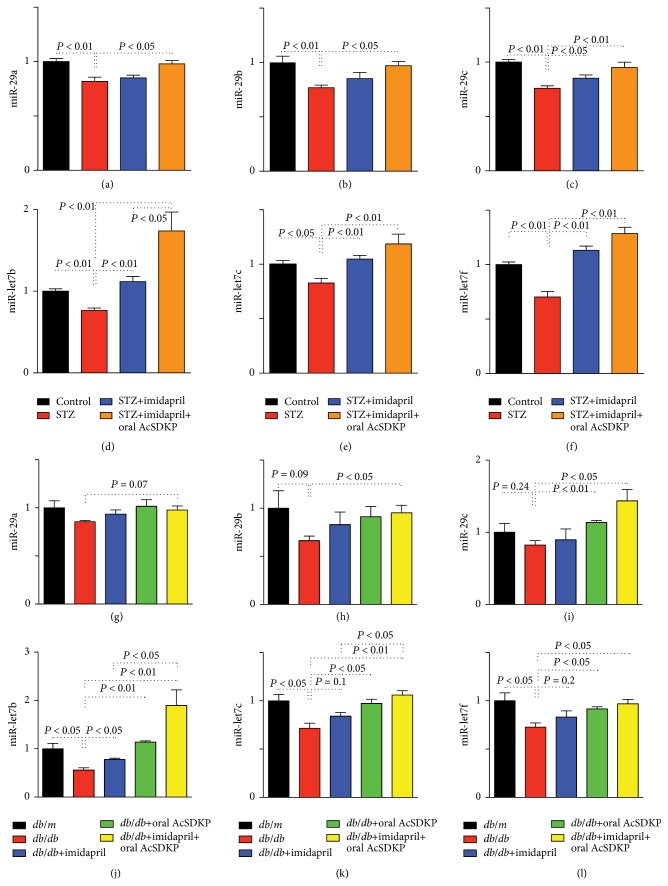
AcSDKP induced antifibrotic miRs. ((a)–(f)) Kidney levels of antifibrotic miR-29s (a–c) and miR-let-7s (d–f) in STZ-induced diabetic CD-1 mice. For the miR-29s, *n* = 4 or 5 were analyzed. For the miR-let-7, *n* = 5 were analyzed. ((g)–(l)) Kidney levels of anti-fibrotic miR-29s (g–i) and miR-let-7s (j–l). For the all miRs, *n* = 3 or 4 were analyzed. The data are expressed as the mean ± SEM in the figure.

We showed that miR- (miR-) 29s and miR-let-7s exhibited antifibrotic effects in diabetic mice [7, 31]. In this study, we again confirmed both miR-29s and miR-let-7s suppression in the STZ-induced diabetic kidney in CD-1 mice; imidapril and imidapril + AcSDKP treatment restored suppressed antifibrotic miRs in diabetic CD-1 mice (Figures 5(a)–5(f)). Additionally, we found that* db/db* mice kidney exhibited a trend of suppression of miR-29s and a significant suppression of miR-let-7s compared to the control* db/m* kidney (Figures 5(g)–5(l)). The intervention groups exhibited a similar trend in which imidapril partially and oral AcSDKP alone nearly normalized levels, and the imidapril + AcSDKP group exhibited much higher levels of these antifibrotic miRs (Figures 5(g)–5(l)). This evidence suggests that these miRs may contribute to the antifibrotic action of oral AcSDKP treatment in* db/db* mice.

### 3.4. Renal Function Was Ameliorated with AcSDKP Treatment in Diabetic Mice

**Figure 6:**
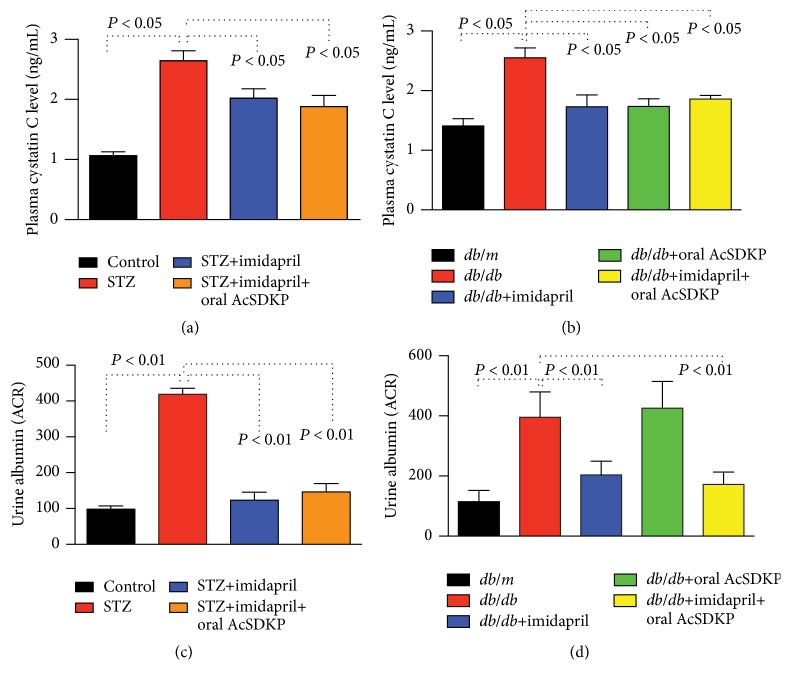
The effects of oral AcSDKP on renal function in diabetic mice. Plasma cystatin C levels (a, b) and urine albumin/creatinine ratios (c, d) in STZ-induced diabetic mice (a, c) and* db/db* mice (b, d) are shown. Cystatin C in CD-1 mice, *n* = 8; in* db/db* mice, *n* = 5 were analyzed. For the urine albumin/creatinine ratio, 4 CD-1 mice and 4 or 5* db/db* mice were analyzed. The data are expressed as the mean ± SEM in the figure.

When evaluated by the plasma cystatin C levels, diabetic CD-1 mice displayed elevated levels of cystatin C, and all the treatments significantly ameliorated renal function (Figure 6(a)). Additionally, the* db/db* mice exhibited higher levels of cystatin C compared to the* db/m* mice. Imidapril, oral AcSDKP, and imidapril + oral AcSDKP combination therapy suppressed plasma cystatin C levels in* db/db* mice, reaching similar levels as those in* db/m* mice, suggesting that these treatments restored impaired renal function in the* db/db* mice (Figure 6(b)). Urine albumin excretion was higher in the diabetic CD-1 mice and* db/db* mice (Figures 6(c) and 6(d)). All interventions with imidapril suppressed urine albumin excretion in the diabetic nephropathy mice (Figures 6(c) and 6(d)). AcSDKP alone did not inhibit urine albumin excretion (Figures 6(c) and 6(d)), similar to the AcSDKP subcutaneous infusion in* db/db* mice [19].

## 4. Discussion

In this study, we confirmed that the antifibrotic peptide AcSDKP may be able to cure diabetic kidney fibrosis using a therapeutic regimen. We preliminarily confirmed that the oral administration of AcSDKP resulted in the elevation of plasma AcSDKP levels in a dose-dependent manner, which was associated with increased AcSDKP levels in the urine. Utilizing both type 1 and type 2 diabetic nephropathy animal models, we confirmed that the oral administration of AcSDKP cured kidney fibrosis and protected renal function.

Kidney fibrosis is the final common consequence of progressive kidney diseases including diabetic nephropathy, resulting in significant damage to the kidney and is associated with deficient kidney function [33–38]. In the process of kidney fibrosis, the suppression of antifibrotic miRs has been well documented. In our current analysis, kidney fibrosis was significantly ameliorated and antifibrotic miR was elevated by the imidapril + oral AcSDKP combination treatment compared to the ACE-I imidapril treatment alone, although cystatin C levels were almost normalized by all treatment groups. This difference may be explained by the sensitivity and power of each analysis and assay system.

Developing an oral pill to cure kidney fibrosis is a significant challenge for both clinicians and scientists. Additionally, diabetic nephropathy is the leading cause of progressive kidney disease from which many patients suffer, resulting in both clinical and financial issues. There have been several attempts to discover oral chemical drugs to target kidney fibrosis in diabetes, such as pirfenidone and bardoxolone methyl [39, 40]. Both drugs have been shown to ameliorate estimated glomerular filtration rates; however, some adverse events are associated with these chemicals [39, 40]. These chemicals are beneficial because they can be easily and affordably produced in large amounts; however, they may be associated with unexpected side effects. AcSDKP is an endogenous peptide associated with the anti-TGF-*β*/Smad signaling pathway [17, 18], anti-PDGF-BB-enhanced mesangial proliferation [29], and anti-inflammation [20]. We have shown that AcSDKP was suppressed in fibrotic STZ mice [14], and herein, we showed the suppression of AcSDKP in* db/db* mice at 24 weeks of age. The restoration of endogenous antifibrotic peptide AcSDKP to normal levels would be safe and physiologically relevant [15]. In our current study, we succeeded in increasing AcSDKP levels to nearly normal levels associated with antifibrotic effects, suggesting that AcSDKP could be a physiological relevant oral drug to cure kidney fibrosis. Additionally, the advantage of using AcSDKP as a drug was clearly revealed by our study, demonstrating that AcSDKP + imidapril can increase the concentration of AcSDKP, suggesting that any ACE inhibitor can control the concentration of AcSDKP.

## 5. Conclusion

We report here that AcSDKP is a drug that exhibits antifibrotic action in diabetic kidney disease in mice. Further studies are required before this attractive antifibrotic molecule AcSDKP can be utilized in clinical medicine. We need to know whether the physiological suppression of AcSDKP is relevant in human diabetic patients with kidney fibrosis. Additionally, whether oral administration of AcSDKP can increase AcSDKP to a therapeutic range in humans would need to be analyzed. Nevertheless, our current study provides hope for diabetic patients with kidney disease. The endogenous antifibrotic peptide AcSDKP may have potential utility in combating diabetic kidney fibrosis via oral administration.

## Supplementary Material

Supplemental Figure 1: Body weight, kidney per body weight, and blood glucose levels in diabetic CD-1 mice.
Supplemental Figure 2: Body weight, kidney per body weight, and blood glucose levels in db/db mice.
