# Supplementary material for: Oral Administration of N-Acetyl-seryl-aspartyl-lysyl-proline Ameliorates Kidney Disease in Both Type 1 and Type 2 Diabetic Mice via a Therapeutic Regimen
Source: Biomed Res Int. 2016 Mar 20;2016:9172157. doi: 10.1155/2016/9172157 (PMC4818806; doi:10.1155/2016/9172157)
Supplement: Supplementary file 1 — Supplemental Figure 1: Body weight, kidney per body weight, and blood glucose levels in diabetic CD-1 mice. Supplemental Figure 2: Body weight, kidney per body weight, and blood glucose levels in db/db mice. [file 9172157.f1.docx]

**
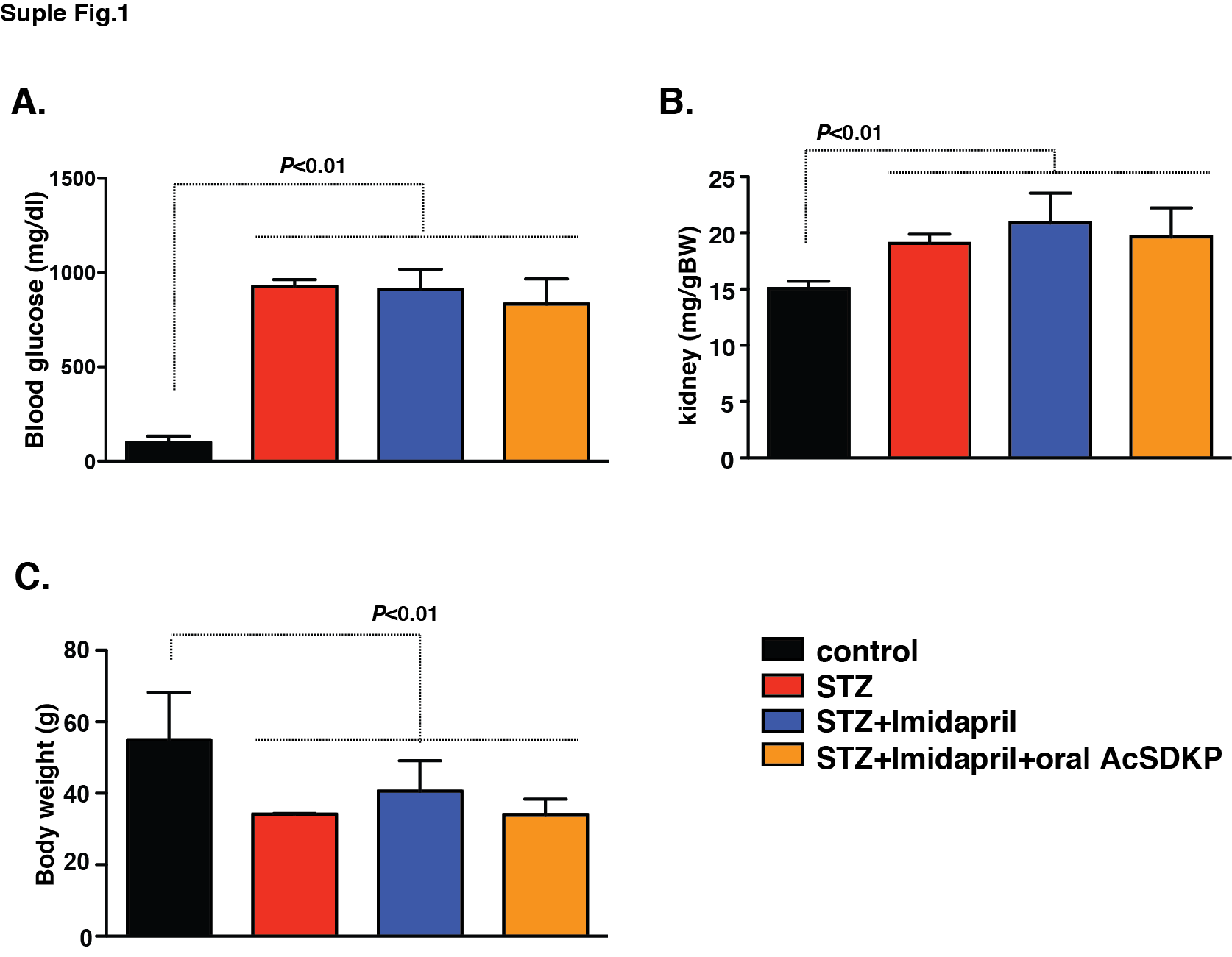
Supplemental Figure 1. Body weight, kidney per body weight, and blood glucose levels in diabetic CD-1 mice.**

Body weight (**A**), kidney per body weight (**B**), and blood glucose levels (**C**) in STZ-induced diabetic mice at 24 weeks after induction of diabetes. Body, kidney per body weight, and blood glucose of CD-1 mice; n=5 were analyzed. For the urine albumin/creatinine ratio, 5 CD-1 mice were analyzed. The data are expressed as the mean + s.e.m. in the figure.

**
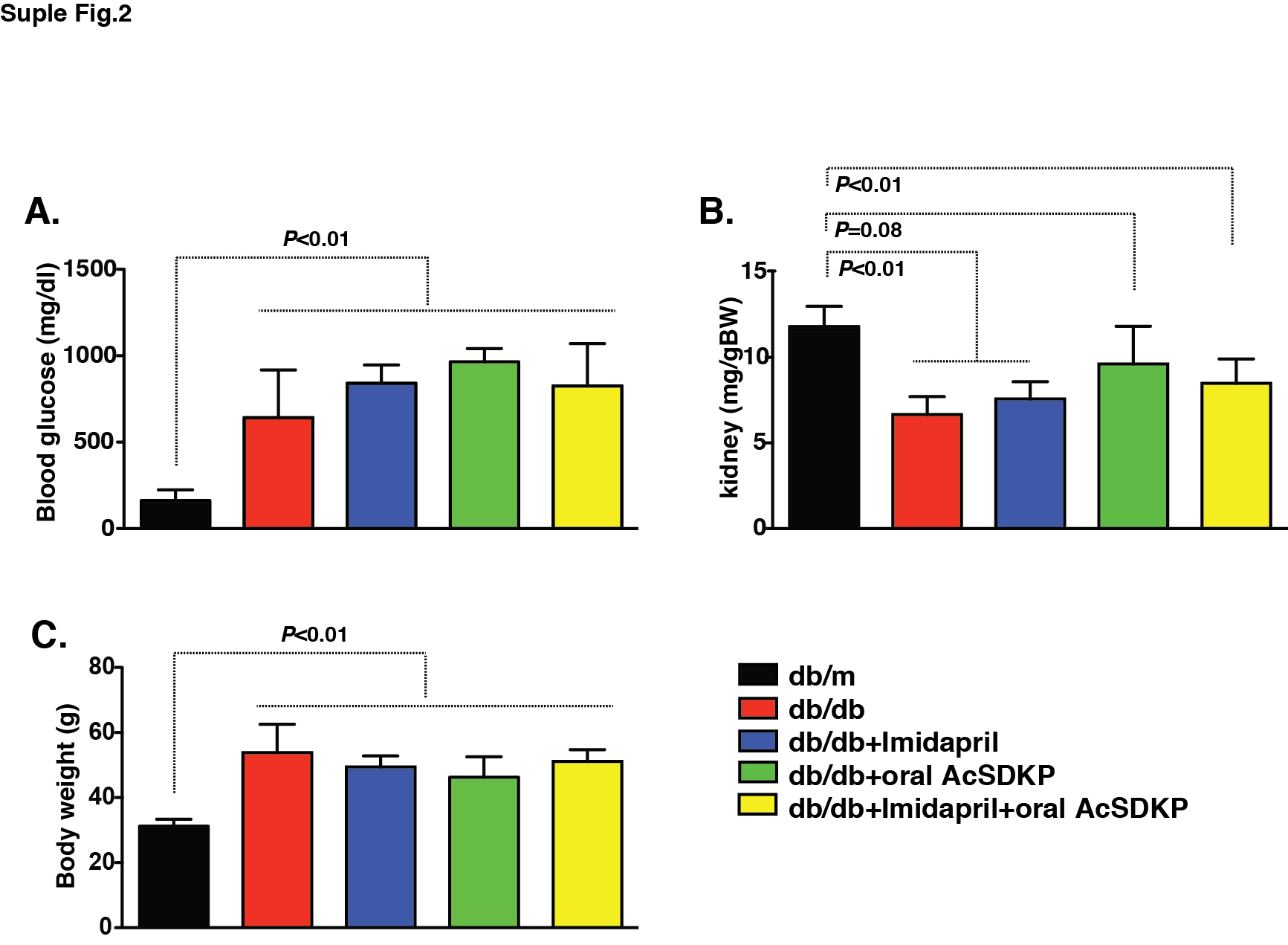
Supplemental Figure 2. Body weight, kidney per body weight, and blood glucose levels in *db/db* mice.**

Body weight (**A**), kidney per body weight (**B**), and blood glucose levels (**C**) at 24 weeks of age in *db/m* or *db/db* mice. Body and kidney per body weight of 5 *db/m* or *db/db* mice were analyzed. For the urine albumin/creatinine ratio, 5 *db/m* or *db/db* mice were analyzed. The data are expressed as the mean + s.e.m. in the figure.
